# In wolves, play behaviour reflects the partners' affiliative and dominance relationship

**DOI:** 10.1016/j.anbehav.2018.04.017

**Published:** 2018-07

**Authors:** Simona Cafazzo, Sarah Marshall-Pescini, Jennifer L. Essler, Zsófia Virányi, Kurt Kotrschal, Friederike Range

**Affiliations:** a Wolf Science Center, Domestication Lab, Konrad Lorenz Institute of Ethology, University of Veterinary Medicine, Vienna, Austria; b Comparative Cognition, Messerli Research Institute, University of Veterinary Medicine, Vienna, Austria; c Department of Behavioral Biology, University of Vienna, Vienna, Austria

**Keywords:** affiliative relationships, dominance relationships, dyadic competitive play, dyadic relaxed play, social assessment

## Abstract

Puppy packs (consisting of only puppies) and mixed-age packs (composed of puppies and adults) were observed to test whether social play can be used for assessing and establishing social relations in wolves, *Canis lupus*. Differently from previous studies, we looked at play behaviours in detail, allowing us to categorize play interactions as either competitive or relaxed, and predicted that different types of play would be associated with different relationships between individuals. We found that the more time dyads spent in relaxed play, the more affiliative interactions they exchanged outside of play. In the mixed-age packs, dyads that spent more time in both relaxed and competitive play showed fewer exchanges of aggressive behaviours. Conversely, in puppy packs, the more time dyads spent in competitive play, the more aggressive interactions were exchanged outside of play. Since clear dominance relationships emerged in the mixed-age packs, but not in puppy packs, we suggest that play can help to reduce the frequency of aggressive interactions only when a clear hierarchy exists between pack members. Furthermore, we found that in both puppy and mixed-age packs, dominance relationships were reflected and rarely reversed during play. Finally, dyads with a less clear dominance relationship spent more time playing in a competitive way. Overall, our results support the social assessment hypothesis suggesting that social relationships outside of play are reflected during playful interactions. Moreover, we revealed how different types of play, that is, playing in a competitive or relaxed way, may be related to different social relationships. This distinction between play types has not been acknowledged before but could help researchers better understand the functions of play in different species.

## Highlights

• Wolves may use play to establish and assess social relationships.
• Play behaviour is correlated with both affiliative and dominance relationships.
• Play may reduce the rate of aggressions when a clear hierarchy exists in the pack.
• Different types of play may be related to different social relationships.

Social play is a widespread phenomenon, suggesting that it may be a critical component of ontogeny. However, little is known about its functional significance despite years of research (reviewed in Burghardt, 2005, Pellis et al., 2015). Given that play most often occurs in juvenile animals, the majority of hypotheses relating to its function have focused on how playing during the immature stage of development fosters the appropriate use of behaviours essential during adulthood or learning about the potential responses of dyadic partners in ‘serious’ contexts (Bugnyar, Schwab, Schloegl, Kotrschal, & Heinrich, 2007). Hence, scientists have mostly concentrated on the delayed benefits of play (Pellis, Pellis, & Bell, 2010). However, play is also common in adulthood in many species (e.g. Cohen, 2006, Cordoni, 2009, Fagen, 1981, O'Meara et al., 2015, Palagi, 2006, Palagi, 2011, Pellis, 2002, Pellis and Iwaniuk, 1999, Pellis and Iwaniuk, 2000a, Pellis and Iwaniuk, 2000b) suggesting that some benefits may be immediate (Breuggeman, 1978, Martin and Caro, 1985, Pellis et al., 2010a, Pellis et al., 2010b, Poirier et al., 1978).

Among others, immediate benefits of play for juveniles and adults may include (1) strengthening of social bonds by increasing trust and reducing aggression between social partners (the social-bonding hypothesis, Bekoff, 1977b, Bekoff, 1977a, Pellis et al., 1992, Soderquist and Serena, 2000) and/or (2) assessment of the competitive abilities of others to establish and maintain dominance relationships without the risks involved in overt aggression (the dominance assessment hypothesis, Dolhinow, 1999, Miller and Byers, 1998). Although both hypotheses have been widely cited as potential functions of social play, only a few studies have empirically tested them.

The social-bonding hypothesis has received some support from a number of studies showing either a correlation between frequency of play and affiliative behaviour (e.g. adult and immature gelada baboons, *Theropithecus gelada,* Mancini & Palagi, 2009; immature Japanese and Tonkean macaques, *Macaca fuscata* and *Macaca nigra*, Petit et al., 2008, Reinhart et al., 2010) or a correlation between an increase in play behaviours and a decrease in aggressive interactions (infant spotted hyaenas, *Crocuta crocuta*, Drea, Hawk, & Glickman, 1996). Conversely, other studies found no evidence supporting an association between social play and both reduced aggressiveness (Sharpe & Cherry, 2003) and increased frequency of affiliative interactions (Sharpe, 2005a).

Contrasting results have also been found for the dominance assessment hypothesis. In a number of primate species, it has been observed that the asymmetry in the exchange of behaviours during play-fighting sessions may reflect the dominance relationship between individuals outside the play context (e.g. Paquette, 1994, Pereira, 1993). In early adolescent boys, Pellegrini (1995) observed a correlation between play fighting and dominance/aggressive displays. Finally, in yellow-bellied marmots, *Marmota flaviventris*, the relative dominance rank calculated by observing the directional outcome of playful interactions in juvenile and yearling marmots correlated significantly with the subsequent dominance ranks calculated from agonistic interactions (Blumstein, Chung, & Smith, 2013). The authors suggested that relationships within play could predict the future dominance relationships outside of play at least in the short term, providing some support for the dominance assessment hypothesis (Blumstein et al., 2013). However, other studies have not found a link between dominance and play. In spotted hyaena cubs, dominance relationships are rigid and established through aggression at an early age. Interestingly, these dominance relationships are ignored, absent or temporarily reversed during play (Drea et al., 1996).

The wolf, *Canis lupus*, is an interesting species in which to investigate the pattern and potential function of social play: wolf packs are characterized by cooperation, high social cohesion and dominance relationships between pack members (Cassidy and McIntyre, 2016, Cassidy et al., 2015, van Hooff and Wensing, 1987; MacNulty et al., 2011, Mech and Boitani, 2003, Packard, 2003, Packard, 2012). Social play may therefore represent an important means of allowing the establishment of dominance relationships in a safe context, potentially reducing aggression and strengthening social bonds to promote cooperation and pack cohesiveness, and of assessing relationships. In this species, social play is common during the juvenile phase (Mech, 1970) and continues into adulthood (Cordoni, 2009). Few studies have been carried out on wolf play behaviour, however, with most focusing on adult individuals (Bekoff, 1995, Bernal and Packard, 1997, Cipponeri and Verrell, 2003, Zimen, 1981, Zimen, 1982). Only one study has investigated the potential validity of the social-bonding and dominance assessment hypotheses in this species (Cordoni, 2009). In a captive group of adult grey wolves, no significant correlations emerged between dyadic play frequencies and affiliative behaviours outside the play context (i.e. body contact and agonistic support frequencies), nor was there a negative correlation between play and aggressive interactions. Nevertheless, play interactions were observed more frequently between partners closest in rank, suggesting that adult wolves may use play to test social partners and as a prelude to contesting rank (Cordoni, 2009). These results would hence provide some support for the dominance assessment hypothesis, but not for the social-bonding hypothesis. Further suggestive evidence for the dominance assessment hypothesis is provided by a recent study investigating play behaviour in wolf pups. Essler et al. (2016) found that pups did not adhere to the 50:50 rule, that is, dyads did not alternate in their winning and losing roles during play, but rather an individual was likely to maintain a constant dominant or submissive role during play with a specific partner. The maintenance of postural asymmetry during play may support the hypothesis that play contributes to the formation of dominance relationships within wolf litters, as has been suggested for other canids (domestic dogs, *C. lupus familiaris*: Scott and Fuller, 1965, Bekoff, 1972; wild red foxes, *Vulpes vulpes*: Meyer & Weber, 1996).

To sum up, previous results on both canids and other species have revealed some correlative support for both the social-bonding and dominance assessment hypotheses. Although we acknowledge that correlative evidence cannot conclusively identify the cause–effect direction between play and social behaviour (Blumstein et al., 2013, Ghiselin, 1982, Sharpe, 2005b), we deem it important to further investigate whether and how behaviours displayed during social play may reflect the partners' affiliative and dominance relationships. Since wolves rely on cooperation between pack members and show relationships moderated according to dominance hierarchies, the social-bonding and dominance assessment hypotheses are not mutually exclusive. Therefore, here we propose a more embracing version of the two hypotheses and suggest that a major function of play in wolves may be social assessment in general; thus, social play may help individuals assess both affiliative and dominance relationships, thereby potentially reducing aggression between pack members (‘social assessment hypothesis’), but also strengthen cooperation. However, we also suggest that different types of play may help individuals assess different types of relationships, as also proposed in previous studies (Bateson and Young, 1981, Biben, 1986, Gomendio, 1988, Martin and Caro, 1985). Sequences of attack, defence and counterattack may characterize a competitive type of play (or play fighting), but sometimes a different form of physical contact between playmates is observed, which includes gentle and friendly behaviours such as pawing and rubbing, resulting in a seemingly relaxed form of contact play. While individuals need to coordinate and modulate their reciprocal behaviours during both types of social play (Bauer and Smuts, 2007, Bekoff, 2001, Biben, 1986, Dugatkin and Bekoff, 2003, Thompson, 1998), it is reasonable to assume that competitive play is better suited to testing the weakness/strength of potential competitive partners and therefore clarifying the reciprocal dominance rank in a potentially safe context (as stated by the dominance assessment hypothesis) and/or decrease the occurrences of aggressive encounters (as stated by the social-bonding hypothesis; Pellis et al., 1993, Pellis and Iwaniuk, 2000b, Palagi, 2006). In contrast, relaxed play should occur mainly between playmates sharing strong affiliative bonds, thus when there is no risk of escalation into aggression. In line with this reasoning, we distinguished between these two types of play to better evaluate their potentially different roles in the social assessment hypothesis.

To test our hypothesis, in the current study we used data on wolf social interactions collected on puppy–puppy and puppy–adult dyads in two consecutive periods: when wolf puppies lived in packs consisting of only puppies (puppy packs) and after their introduction into previously established packs of adult wolves (mixed-age packs).

Based on all the considerations for variation in the form and function of social play given above, some specific predictions were tested.

In particular, according to the social-assessment hypothesis, social play may be associated with both a low frequency of aggressive encounters and a high frequency of affiliative interactions. Therefore, based on our previous assumption about competitive and relaxed play, we predicted that dyads spending more time in both relaxed and competitive play should engage in fewer aggressive interactions (prediction 1). Furthermore, dyads spending more time engaged in relaxed play should engage in more affiliative interactions outside the play context (prediction 2).

Play interactions may also help to establish and/or maintain dominance relationships outside the play context. Accordingly, we would expect to find a positive correlation between the frequency and direction of competitive behaviours displayed during dyadic play and the frequency and direction of rank indicator behaviours (i.e. submissive and/or dominant behaviours) displayed during dyadic encounters occurring outside the play context (prediction 3). Furthermore, clear dyadic hierarchical relationships entail a high degree of asymmetry or even a complete unidirectionality in the relationship (e.g. de Vries, 1995). If competitive play helps individuals to establish a hierarchy outside play, then dyads with a clear rank relationship should play in a competitive way less than dyads with a more symmetric/unclear relationship. Therefore, on the one hand, we would expect to find a positive correlation between the duration of dyadic competitive play and the equity/symmetry in the exchange of rank indicator behaviours (prediction 4a), and on the other hand, we would not expect to find any correlation between the duration of dyadic relaxed play and the equity/symmetry in the exchange of rank indicator behaviours (prediction 4b).

Finally, since previous studies in a number of species have shown that playmates' age and sex (dogs: Bauer and Smuts, 2007, Pal, 2010; wolves: Essler et al., 2016; hyaenas: Drea et al., 1996; gorillas, *Gorilla gorilla*: Brown, 1988, Maestripieri and Ross, 2004, Palagi et al., 2007; rats, *Rattus norvegicus*: Pellis et al., 1997, Foroud and Pellis, 2002; wallabies, *Macropus rufogriseus banksianus*: Watson & Croft, 1996; baboons, *Papio cynocephalus*: Pereira, 1984; bonobos, *Pan paniscus*: Palagi, 2006; squirrel monkeys, *Saimiri sciureus*: Biben, 1986) as well as their relatedness (macaques, *M. fuscata*: Glick, Eaton, Johnson, & Worlein, 1986; primates: Berman, 2004) may affect patterns of play, we also took these variables into account when testing our hypothesis.

## Methods

### Subjects and Study Site

**Figure 1:**
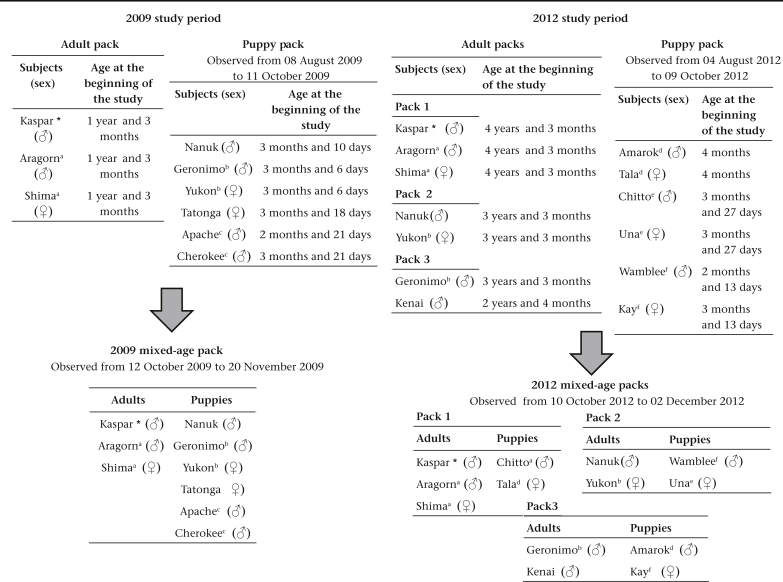
Distribution of subjects within the packs for both study periods. Matching lowercase letters denote siblings. Asterisks denote cousins.

The subjects and study site have been described in detail by Essler et al. (2016). We observed two packs of wolf, *C. l. occidentalis*, puppies from 3 to 5 months of age at the Wolf Science Center (WSC, Ernstbrunn, Austria) in 2009 and 2012, respectively (Fig. 1). Both packs consisted of six puppies: the pack observed in 2009 (2009 puppy pack, hereafter) was composed of two pairs of siblings and another two unrelated individuals while the pack observed in 2012 (2012 puppy pack, hereafter) was composed of three pairs of siblings. Therefore, both kin and nonkin individuals were present in each pack (see Fig. 1).

The wolves that participated in this study originated from North America but were born in captivity. All of them were hand-raised in peer groups by professional WSC staff after being separated from their mothers during the first 10 days after birth. Therefore, for the first 4 months of their life the puppies lived in a single pack in an enclosure spending all their time in the presence of humans (for more information on raising methods, see Range and Virányi, 2013, Range and Virányi, 2014).

Afterwards, all of them were introduced into previously established packs of adult wolves. In particular, in 2009, the six puppies (four males and two females) were integrated into a previously established group of three adult wolves (two males and one female) from the 2008 litter. The resulting pack of nine individuals was analysed for play interactions between adult and juvenile wolves (2009 mixed-age pack, hereafter). The six wolf puppies of 2012 (three males and three females) were integrated into three different, previously established packs of adult wolves from the 2008, 2009 and 2010 litters. The resulting mixed-age packs of nine individuals in 2009 (2009 mixed-age pack, hereafter), and of five, four and four individuals, respectively, in 2012 (2012 mixed-age packs, hereafter) were analysed for play interactions between adult and juvenile wolves. To see the details of the packs observed for each study period as well as information of related individuals see Fig. 1.

Both puppy and mixed-age packs were housed in enclosures (approximately 4000–10 000 m^2^) consisting of large, fenced outdoor areas, raised platforms for shelters, as well many bushes, trees and sometimes fallen tree trunks. The animals were fed with pieces of meat, fruits, milk products and dry food. During the first few months of their lives, they were fed several times per day, which was slowly reduced to only twice per week, in accordance with dietary requirements. Drinking water was permanently available.

### Ethical Note

This study was purely observational with no manipulation of animals. The relevant committee, Tierversuchs-kommission am Bundesministerium für Wissenschaft und Forschung (Austria) allows us to run this research with no special permissions regarding animals (wolves) (Tierversuchsgesetz 2012 – TVG 2012).

### Data Collection

We video recorded social interactions (i.e. play, agonistic and affiliative interactions) using DCR-SR35 Sony digital video cameras. Data were collected from the beginning of August 2009 until the end of November 2009 (2009 observation period) and from the beginning of August 2012 until the end of December 2012 (2012 observation period). All observations were equally distributed across the daytime between 0600 and 2000 hours.

The focal animal sampling method (Altmann, 1974) was used to collect two different types of behavioural data: play observations, which were used to code play behaviours, and general observations, which were used to code affiliative and agonistic (aggressive, dominant and submissive behaviours) interactions.

#### Play observations

For the aim of the current study, we used only dyadic play bouts. A play bout was considered to start when one wolf directed a playful behaviour towards its playmate and ended when the participants stopped their behaviours or one of them moved away (see Essler et al., 2016 for further details).

In total we obtained 8.1 h and 7.5 h of video for the 2009 and 2012 puppy packs, respectively, and 5.7 h and 5.9 h of video for the 2009 and 2012 mixed-age packs, respectively.

Some of the videos used for this study were recorded in the framework of a master thesis by Heufelder (2010) and recoded for the purposes of this study.

#### General observations

General observations were collected to determine the frequency and direction of aggressive, dominance, submissive and affiliative behaviours between individuals outside of play. All observations were carried out using the focal animal sampling method (10 min of recording, Altmann, 1974). More detailed information about data collection are reported in Essler et al. (2016).

### Behavioural Coding

Video recordings of the play sessions and the general observations were coded in the program Observer XT 11.0 (Noldus Information Technologies, Wageningen, The Netherlands). All videos were coded by J.E. and S.C., except videos for the 2009 general observation data, which were coded by Teresa Schmidjell. Twenty per cent of videos of play observations were coded independently by the two main observers (S.C. and J.E.) to obtain interobserver reliability coding. An overall Kappa coefficient (Bakeman and Gottman, 1986, Cohen, 1960) covering all play behaviours and play partner identities was calculated and averaged 0.78.

#### Play coding

For all play videos, each session of dyadic play involving the focal animal and lasting at least 5 s was coded. Furthermore, we also coded all other dyadic play sessions that were visible on the video and lasted at least 5 s, but did not involve the original focal individual. For all dyadic play sessions, we coded the identity of the two playmates, the duration of the play session and all play behaviours displayed by both playmates.

To code all play behaviours, we adopted the same ethogram used by Essler et al. (2016), although for the aims of the current study, the play behaviours were separated into two different types of interactions: competitive play and relaxed play behaviours. Competitive play behaviours included offensive behavioural patterns and self-handicapping behavioural patterns. Offensive behaviours were defined as those used by individuals to gain a winning position (Essler et al., 2016); they include behaviours such as biting and chasing as well as mounting and standing over the partner. Self-handicapping behaviours were defined as those used by individuals to give up a winning position to their partner such as lying on the back as well as being physically under their partner (Essler et al., 2016). Finally, relaxed play was coded when both playmates engaged in play interactions not involving any offensive or self-handicapping behavioural patterns. For a detailed description of play behaviours see Table 1.

**Table 1:** Ethogram of play behaviours

Behaviours		Description
**Competitive play behaviours**		
Offensive behaviours		
	Bite shake	A bites R and shakes head back and forth while maintaining a hold on R
	Play bite	A gives an inhibited bite to R (without shaking the head)
	Chase/charge	A runs after R with a least two running strides while R runs or trots away from A, or A breaks from a stalking position into a run, moving directly towards R
	Chin over	A places the underside of its chin over R's back, usually right behind the neck or near R's shoulders, but sometimes over R's head
	Paw on	A stands up on its hindlegs and puts front legs on R's shoulders, usually silent and with open mouth; individuals can bite each other
	Forced down	A uses physical force or contact to cause R to drop completely to the ground from a moving, standing or sitting position. Force may be applied with a bite (pin), push/tackle, body slam, bouncing into him (knock down) or some other forceful movement
	Mount (push/tackle)	A rears up (keeping hindlegs on the ground) to place forelegs on R's back. A has a rounded spine with curved front legs and forepaws to grasp R's torso. Pelvic thrusting may or may not be present (if it results in a down, it was coded as forced down instead of independent push/tackle)
	Muzzle bite	A places mouth around R's muzzle
	Over	A sits on, stands over or lies over R with at least 25% of A's torso over R's torso
	Overs during downs	A stands over or lies on R with at least 50% of A's torso over R's torso (or vice versa: 50% of A's torso is under R's torso), or A sits and exerts weight directly on R's head or torso with a distinct pause in the sitting position
Self-handicapping behaviours		
	Muzzle lick	A licks on or around R's muzzle. A lick may or may not be accompanied by nudging
	Receive genital sniff	A holds hindlegs apart while in belly-up position on the ground to allow R to put snout on or near A's genitals for an investigatory sniff
	Voluntary down	A drops completely to the ground from a moving, standing or sitting position without R's physical enforcement. R and A must be interacting when A goes down
	Unknown down	Definition same as ‘voluntary down’; however, owing to the camera angle, it is unclear whether the down is forced or voluntary, but a definite asymmetry in positions exists
**Relaxed play behaviours**		
		Both A and R are sitting or lying on the ground pawing each other and rubbing against each other. These behaviours are often accompanied by a ‘play face’ (relaxed open mouth). Neither individual displays a ‘winning’ position over the other and none of the competitive behaviours described above are displayed by either playmate

All behaviours coded in the play context (from Essler et al., 2016) are listed and described. A: wolf performing the behaviour; R: wolf receiving the behaviour.

Dyadic play was only coded when both individuals were engaged in play, and not when one individual was merely persistent in play invitations. Because we did not always record the beginning of the play bout, play invitations were not used to analyse any playmate preferences. Play bouts of the same play partners recorded during a single filming session were considered independent if they were separated by a minimum of 15 s of nonplay behaviour. To ensure that we did not skew the data with dyads that played only for a few seconds over the course of the entire study period, we only included dyads that played for at least 1 min (all bouts added).

#### General observation coding

Although for each general observation video, one focal subject was followed at a time, we coded all nonplay interactions between individuals visible in the video even if they did not interact with the focal individual. This allowed us to increase our number of nonplay interactions in the analyses. In particular, we coded all occurrences of aggressive, submissive and dominance interactions as well as affiliative interactions. We also recorded the duration of the visibility of each subject in the video to calculate behaviours in relation to the time an animal was observed to correct for differences in visibility. The social behaviours used to code our general observations are summarized in Table A1 (see Appendix), including the behaviours used as rank indicators.

### Data Analyses

To characterize the social relationship of each dyad for all packs/periods we calculated (1) the frequency of affiliative and aggressive behaviours exchanged in each dyad, (2) the frequency of behaviours used to calculate rank (rank indicator behaviours) and competitive play behaviours displayed by each dyad member towards its partner, (3) the asymmetry in the exchange of behaviours used to calculate rank in each dyad, and (4) the duration of dyadic competitive and relaxed play. These different types of measures are detailed below.

The frequencies of affiliative and aggressive behaviours were calculated by adding separately all affiliative behaviours and aggressive behaviours (see Table A1 for a description) that individual a showed towards b and b towards a, normalized for the observation time of each dyad.

The frequency of rank indicator behaviours was calculated for both members of each dyad by adding the number of dominance behaviours displayed to the number of submissive behaviours received. Aggressive behaviours were excluded from this frequency since in a previous study on the same packs we found that linear dominance relationships were well described by dominance and submissive behaviours but not by aggressive interactions (Essler et al., 2016). The frequency of competitive play behaviours was obtained for both individuals of each dyad by adding the number of offensive behaviours displayed to the number of self-handicapping behaviours received.

We assessed whether rank indicator behaviours (i.e. dominance behaviours displayed plus submissive behaviours received) were balanced or skewed in nonplay relationships by calculating the dominance relationship asymmetry (Aab) index (adapted from Mitani, 2009 and Silk, Altmann, & Alberts, 2006). The Aab was calculated in terms of the proportion of behaviours that individual a showed towards individual b minus the proportion of behaviours that individual b showed towards individual a. Then we took the absolute difference of this quantity and subtracted it from 1, according to the following formula:

$$\mathrm{Eab}=1-\left|\frac{Ba\to Bb}{Ba↔Bb}-\frac{Bb\to Ba}{Ba↔Bb}\right|$$

where Ba→Bb is the total number of behaviours that a directed at b, Bb→Ba is the total number of behaviours that b directed at a, and Ba↔Bb is the total number of behaviours exchanged between a and b. Relationship equity scores ranged from 0 indicating that the behaviour was completely skewed to 1 indicating that the behaviour was completely equitable.

Finally, to assess whether the amount of time that two individuals spent playing affected their affiliative and aggressive relationships, we took the type of play into consideration by estimating the proportion of time each dyad spent playing in a competitive or a relaxed manner.

#### Test models

To test our predictions, we used linear mixed-effects models (LMMs) and generalized linear mixed-effects models (GLMMs). We tested whether model assumptions (i.e. normally distributed residuals and homogeneity of variances for LMMs and overdispersion for GLMMs) were fulfilled and accounted for if necessary by running the GLMM model using a penalized quasilikelihood (PQL) approach. As individuals were parts of multiple dyads, and each dyad could occur more than once if they were both part of the same puppy and mixed-age packs, we included dyad (and individual where applicable) as a random factor to avoid pseudoreplication. We also included age category combination (puppy–puppy, puppy–adult), sex combination (male–female, male–male, and female–female), kin relationship (kin, nonkin) and pack type (puppy and mixed-age packs) as control variables where applicable. We used a backward stepwise reduction procedure based on *P* values (Grafen & Hails, 2002) to avoid problems due to inclusion of nonsignificant terms (Engqvist, 2005). Variables employed for each model are available in Table A2, Table A3, Table A4, Table A5, Table A6 (see Appendix). All model analyses were performed using R v3.2.5 (The R Foundation for Statistical Computing, Vienna, Austria, http://www.r-project.org). We implemented LMMs using the ‘lmer'function of the'lmerTest'package (Kuznetsova, Brockhoff, & Christensen, 2016) and GLMMs using the'glmer'function in the ‘lme4'package (Bates, Maechler, Bolker, & Walker, 2015); the ‘glmmPQL’ function was fitted using the ‘nlme’ (Pinheiro, Bates, DebRoy, Sarkar, & R Core Team, 2016) and ‘MASS’ (Venables & Ripley, 2002) packages (R Development Core Team 2016).

#### Models 1 and 2

To test whether there was a relationship between the time spent playing by a dyad and the frequency of aggressive (prediction 1, model 1) and affiliative (prediction 2, model 2) interactions outside of play, we ran a GLMM with a Poisson distribution, with either the frequency of aggressive or affiliative behaviours as the response variables (normalized by observation time by including the offset function in the model formula), and competitive play duration, relaxed play duration, age and sex combination, kin relationship and the interactions between both competitive and relaxed play durations and pack type as predictor variables. The latter interactions were included in the analyses since, considering puppies spend more time in play than adults, play durations may affect the response variable differently if the pack is composed only of puppies (puppy packs) or both puppies and adults (mixed-age packs).

#### Model 3

To investigate whether the frequency and direction of competitive play behaviours in a dyad are reflected in the frequency and direction of rank indicator behaviours displayed during nonplay interaction (prediction 3), we ran a GLMM, with the frequency of rank indicator behaviours (normalized by observation time by including the offset function in the model formula) as the response variable and the frequency of competitive play behaviours (normalized by observation time), age and sex of both actors and receivers and kin relationship as predictor variables considering only dyads in which both individuals were observed playing.

#### Model 4

To test whether the asymmetry in the exchange of rank indicator behaviours reflects the duration of competitive play (prediction 4a) or relaxed play (prediction 4b), we ran an LMM with either competitive play duration (model 4a) or relaxed play duration (model 4b) as the response variable and the dominance relationship asymmetry index, age and sex combination, kin relationship and the interaction between dominance relationship asymmetry index and pack type as predictor variables. The asymmetry index*pack type interaction was included since puppy–puppy dyads have more equal play than puppy–adult dyads (see Essler et al., 2016). Hence the dominance relationship asymmetry index may affect the response variable differently if the packs are composed only of puppies (puppy packs) or both puppies and adults (mixed-age packs).

### Results

An overview of the results is reported in Table 2.

**Table 2:** Summary of predictions and results

Predictions	Results
Prediction 1. Dyads spending more time in relaxed and competitive play should engage in fewer aggressive interactions outside the play context	Confirmed for mixed-age packs but not for puppy packs
Prediction 2. Dyads spending more time in relaxed play should engage in more affiliative interactions outside the play context	Confirmed for mixed-age packs and only partially for puppy packs
Prediction 3. The frequency and direction of competitive behaviours displayed during dyadic play should be positively correlated with the rank indicator behaviours displayed during dyadic conflicts occurring outside the play context	Confirmed
Prediction 4a. The dyadic competitive play duration should be positively correlated with the equity/symmetry in the exchange of rank indicator behaviours	Confirmed
Prediction 4b. No correlation should be detected between dyadic relaxed play duration and the equity/symmetry in the exchange of rank indicator behaviours	Confirmed

### Prediction/Model 1

**Figure 2:**
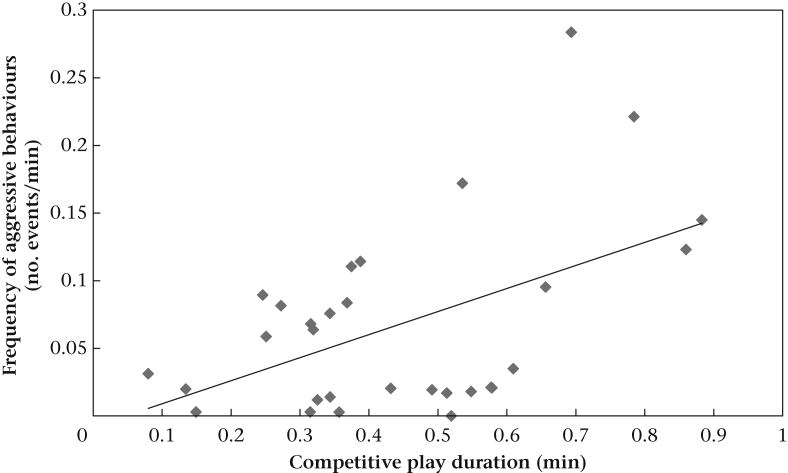
Relation between the frequency of aggressive behaviours and competitive play durations in puppy packs.

Model 1 concerned the relationship between the time spent playing by a dyad and the frequency of aggressive interactions outside of play. After a backward stepwise reduction, we found a significant interaction between both the duration of competitive play and pack type (puppy and mixed-age packs) and the duration of relaxed play and pack type (Appendix Table A2). Therefore, we ran separate models for puppy and mixed-age packs. In puppy packs, the longer a dyad spent in competitive play, the more aggressive interactions were observed outside of play (Appendix Table A2, Fig. 2). Furthermore, sibling pairs exchanged fewer aggressive interactions than nonsibling pairs (Appendix Table A2). There was no relationship between the duration of relaxed play and the frequency of aggressive behaviours in a dyad (Appendix Table A2).

**Figure 3:**
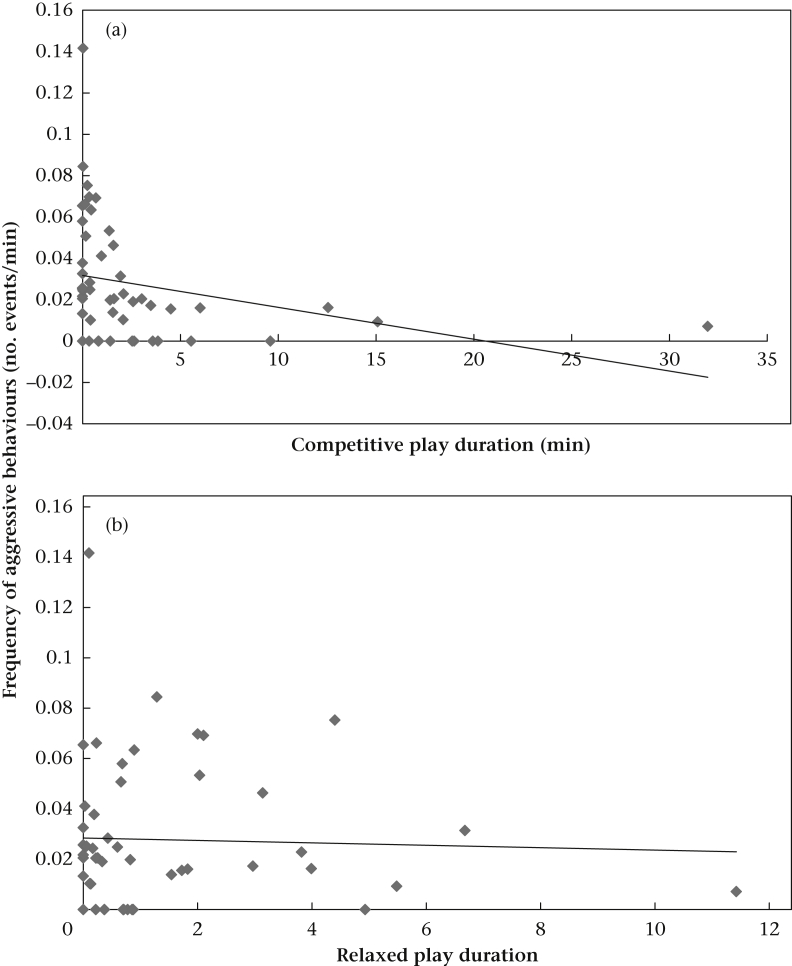
Relation between the frequency of aggressive behaviours and both (a) competitive and (b) relaxed play durations in the mixed-age packs.

Conversely, in the mixed-age packs, the more time each dyad spent in both competitive play and relaxed play, the fewer aggressive interactions were exchanged outside of play (Appendix Table A2, Fig. 3). Since in the mixed-age packs we had very few kin dyads (*N* = 3) we did not include kinship as a predictor variable in this model.

### Prediction/Model 2

In model 2 we considered the relationship between the time spent playing by a dyad and the frequency of affiliative interactions outside of play.

**Figure 4:**
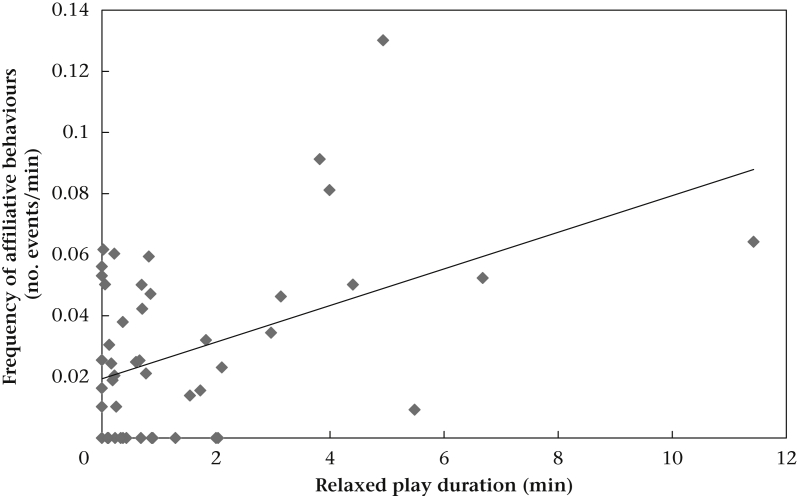
Relation between the frequency of affiliative behaviours and relaxed play durations in the mixed-age packs.

After a backward stepwise reduction, we found a significant interaction between the duration of relaxed play and pack type (Appendix Table A3). Conversely, neither competitive play nor its interaction with pack type was significant and neither were all other predictor variables (Appendix Table A3). We hence ran separate models for puppy and mixed-age packs with relaxed play duration as the only predictor variable. For puppy packs, we found only a nonsignificant tendency towards a positive correlation between relaxed play duration and frequency of affiliative interactions (Appendix Table A3). In the mixed-age packs, the relationship emerged clearly: the longer a dyad spent in relaxed play the more affiliative interactions were observed outside play (Appendix Table A3, Fig. 4).

### Prediction/Model 3

**Figure 5:**
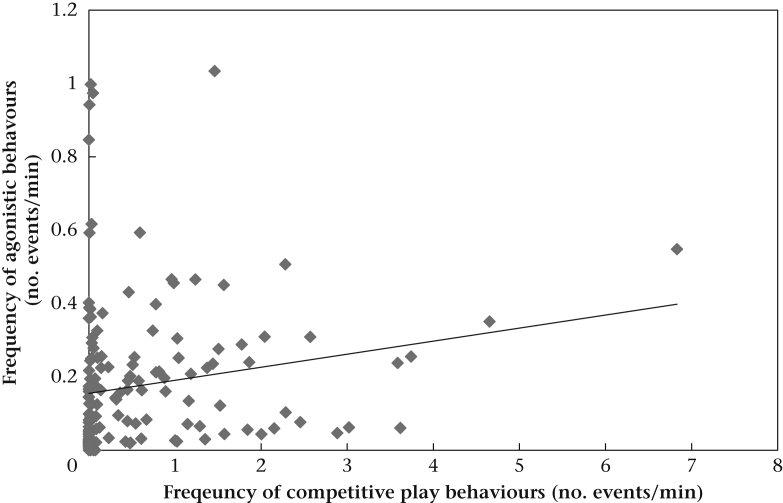
Relation between the frequency of rank indicator behaviours displayed outside the play context and the frequency of competitive behaviours displayed during play for all packs.

In model 3 we considered the relationship between the frequency and direction of competitive behaviours displayed during competitive play in a dyad and the frequency and direction of rank indicator behaviours displayed during nonplay interactions. After model reduction, we found a positive relationship between the frequency and direction of competitive play behaviour and the frequency and direction of rank indicator behaviours (Appendix Table A4, Fig. 5). Thus, the frequency and direction of competitive play behaviours exchanged by playmates during play bouts reflect the frequency and direction of rank indicator behaviours exchanged by dyad members during nonplay interactions. In other words, in each dyad the subject displaying more dominant behaviours towards and receiving more submissive behaviours from its partner during agonistic interactions occurring outside the play context also displays more offensive behaviours towards and receives more self-handicapping behaviours from the same partner during play. We also found a significant effect of age of both the actor and the receiver, with adults showing more rank indicator behaviours than puppies and puppies receiving more rank indicator behaviours than adults (Appendix Table A4). The sex of the actor also had an effect with males displaying more rank indicator behaviours than females (Appendix Table A4).

### Prediction/Model 4a

Model 4a concerned the relationship between the asymmetry in the exchange of rank indicator behaviours and the duration of competitive play.

**Figure 6:**
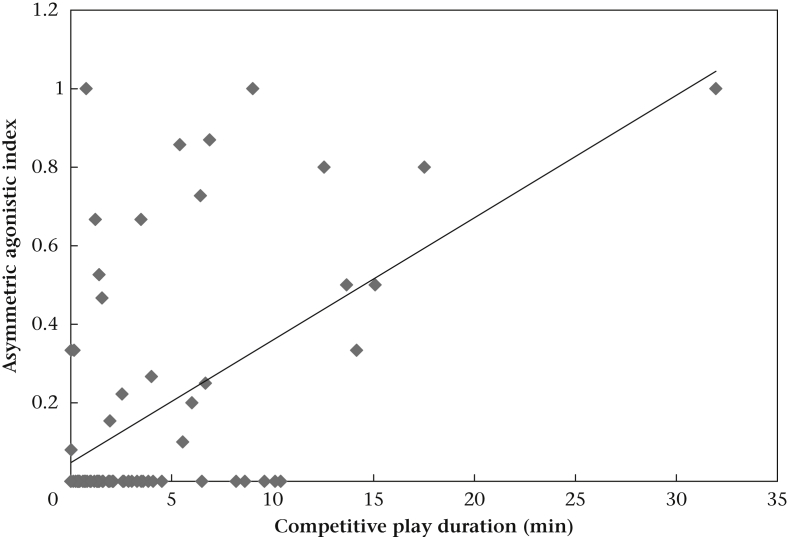
Relation between the dominance relationship asymmetry index and the duration of competitive play for all packs.

We did not find an interaction between the asymmetry in the dominance relationship and pack type (Appendix Table A5). After model reduction, we found that dyads with a more symmetrical exchange of rank indicator behaviours spent more time engaged in competitive play (Appendix Table A5, Fig. 6). We also found an effect of kinship with kin dyads spending more time in competitive play than nonkin dyads (Appendix Table A5).

### Prediction/Model 4b

Model 4b concerned the relationship between the asymmetry in the exchange of rank indicator behaviours and the duration of relaxed play.

We did not find an interaction between the asymmetry in the rank indicator relationship and pack type (Appendix Table A6). After model reduction, we found that the asymmetry of dominance relationships, sex combination, age combination, kinship and pack type (Appendix Table A6) had no effect on the duration of relaxed play.

## Discussion

Overall, our results provide correlative evidence in support of the social-assessment hypothesis. Furthermore, they clearly support the distinction between different types of social play (competitive and relaxed) since these were found to correlate differently with patterns of behaviours occurring outside of play.

In agreement with the social-assessment hypothesis, the more time dyads in the puppy packs spent in relaxed play, the more affiliative interactions they exchanged outside of play, though the relationship was not significant (prediction 2 not completely supported, Table 2). Furthermore, when puppies were integrated into groups of adult wolves, the results also supported the social-assessment hypothesis for both puppy–puppy and puppy–adult dyads. In fact, once in their final packs, dyads spending more time in both relaxed and competitive play showed fewer exchanges of aggressive behaviours (prediction 1 supported, Table 2), and dyads spending more time just in relaxed play also showed more frequent exchanges of affiliative behaviours (prediction 2 supported, Table 2). These results suggest that, regardless of the type of play, wolves play more when they have no issues with one another but playing in a relaxed way is mainly seen between individuals sharing good relationships. However, contrary to what we expected, in puppy packs the more time each dyad spent in competitive play, the more aggressive interactions were exchanged outside of play (prediction 1 not supported, Table 2). This positive correlation between competitive play and aggressive behaviours found in puppy packs may be because, whereas clear dominance relationships emerged in the mixed-age packs, in puppy packs these relationships were still rather undefined suggesting that a clear hierarchy was not yet fully established (Essler et al., 2016). Indeed, it may be argued that it is only after the establishment of clear dominance relationships that play can modulate aggression by strengthening social affiliative bonds and help to reduce the frequency of aggressive interactions (as found in spotted hyaenas; Drea et al., 1996). Accordingly, in the mixed-age packs, where the hierarchy was clear, we found a positive correlation between relaxed play duration and the frequency of affiliative interactions (Fig. 4) and a negative correlation between both relaxed and competitive play duration and the frequency of aggressive interactions (Fig. 3). From this perspective, play in wolves may help juveniles establish close social bonds with their pack members, allowing for future cooperative interactions in hunting, territorial defence and pup rearing.

Taken together, these results suggest, first, that the correlation between play behaviours and social relationships may change according to the social environment (puppy packs versus mixed-age packs); second, they reveal how different types of play, that is, playing in a competitive or relaxed way, may be related to different social relationships.

Our findings are in contrast with previous results on adult wolves: Cordoni (2009) found that there was no relationship between play frequency and affiliative relationships and level of aggression. However, there were several methodological differences between the studies. First, our analyses concerned puppy–puppy and puppy–adult dyads in several packs, whereas Cordoni (2009) analysed a single pack of adult wolves; second, our packs were composed of related and unrelated individuals, while Cordoni's pack was a disrupted family (sensu Packard, 2003), in which the alpha female was missing. Furthermore, instead of frequencies, we analysed play durations, taking into consideration two different types of play (competitive and relaxed play). Finally, we assessed the affiliative relationship based on the exchange of several affiliative behavioural patterns (see Appendix Table A1), while Cordoni (2009) based this measure on body contact and agonistic support frequencies. Although all these factors (particularly age group differences) may have played a role in the differences observed between studies, it is of particular interest that the distinction of different types of play allowed us to detect a relationship with other social variables (i.e. aggression and affiliation) that would not have emerged had we considered play as a unified behavioural category. Therefore, these results highlight the importance of investigating play function in different periods of ontogeny (i.e. during immature and adult phases), taking the different types of play and social relationships into account.

Predictions relating to the dominance assessment hypothesis (predictions 3 and 4, Table 2) were also supported by our results. First, the direction and frequency of competitive play behaviours displayed in each dyad clearly reflected the frequency and direction of rank indicator behaviours (i.e. dominance behaviours displayed plus submissive behaviours received) occurring outside the play context (prediction 3 supported, Table 2), and this was the case in both puppy and mixed-age packs. The frequency and direction of rank indicator behaviours have been used to assess dominance relationships in our packs (Essler et al., 2016). Hence, dominance relationships were reflected and not reversed during play as has previously been found in primates (Pereira, 1993; Paquette, 1994). Second, we found that, regardless of group composition, dyads with a more symmetric exchange of rank indicator behaviours, thus with a less clear dominance relationship, spent more time playing in a competitive way (prediction 4a supported but not prediction 4b, Table 2) suggesting that they might be using play to help them clarify their dominance relationship. Based on these results, we suggest that play in wolves may be used to establish and maintain dominance relationships as suggested for a number of other species (humans: Smith and Boulton, 1990, Pellegrini, 1995; primates: Paquette, 1994, Palagi, 2006; rats: Smith, Fantella, & Pellis, 1999; canids: Scott and Fuller, 1965, Bekoff, 1972, Meyer and Weber, 1996; but see Pellis et al., 1993). Note that in our packs, puppy–puppy dyads did play more equally than puppy–adult dyads (Essler et al., 2016). Therefore, our current results indicate that the asymmetric competitive play relationships of puppy–adult dyads as well as the symmetric competitive play relationships of puppy–puppy dyads are both mirrored within the agonistic context.

Our finding that wolves spent longer playing with partners with whom they need to clarify their dominance relationship is in accordance with Cordoni's (2009) result that, in adult wolves, rank distance between conspecifics was negatively correlated with play distribution. This was interpreted as an indication that by playing with conspecifics closest in rank position, wolves may test each other to acquire information about the skills of potential competitors and gain hierarchical advantage over them. In line with that, we found that siblings, which were in most cases close to each other in rank, spent more time engaged in competitive play. Although we cannot conclusively establish the causal link between play and dominance, taken together our results show the existence of clear relationships between these variables, with the same dominance relationship being reflected within and outside play.

In the current study, we found no sex differences in either competitive or relaxed play (see models 4a and 4b) probably because in wolf society, as in other canid species, males and females share similar roles and behavioural repertoires (e.g. Mech, 1995, Mech, 1999, Mech et al., 1999, Packard et al., 1992, Peterson et al., 2002). In line with this, the absence of sexual dimorphism in play has also been found in other studies of several canid species (e.g. Bekoff, 1974, Biben, 1983, Cordoni, 2009).

Overall, our results support most of the predictions generated from the social-assessment hypothesis, which includes and reconciles both the social-bonding and the dominance assessment hypotheses. Therefore, playful activity in wolves may have an important role in social assessment as has been observed in a number of other primate and nonprimate species (e.g. Pellis and Iwaniuk, 1999, Pellis and Iwaniuk, 2000a, Pellis and Iwaniuk, 2000b, Drea et al., 1996, Mancini and Palagi, 2009, Palagi, 2006, Palagi et al., 2004). Nevertheless, although in children more relaxed and noncompetitive play interactions have received considerable attention (e.g. Pellegrini, 2011), most of the previous studies on nonhuman animals have focused on the analysis of just competitive play (often referred to as ‘play fighting’ or ‘rough-and-tumble play’). Only a few studies on bonobos and chimpanzees, *Pan troglodytes*, have investigated the differences between competitive and relaxed play (referred to as ‘gentle play’, see e.g. Paquette, 1994, Palagi, 2006, Palagi and Paoli, 2007), but just in terms of differences in the frequency of occurrence in relation to the sex of playmates. To our knowledge, no previous study has analysed the relationship between these two different types of social play and social interactions characterizing the dyad's relationship outside of play. Indeed, our results support the distinction between these two types of social play since both competitive and relaxed play duration was correlated with the frequency of aggressive interactions, while only relaxed play duration was correlated with the frequency of affiliative interactions. Also, competitive play duration, but not relaxed play duration, was related to the asymmetry of dominance relationships. Nevertheless, although we analysed two distinctive forms of social play, we cannot exclude the possibility that these are not distinct forms of play but are part of a continuum from one form to the other. In other words, the intensity of social play may vary along a gradient, with ‘relaxed’ play at one extreme and ‘competitive’ play at the other, as suggested in other studies (e.g. Pellis, 1981). Whether relaxed and competitive play encounters are effectively different in their form or degree may be particularly relevant for the study of the mechanisms of social play (see for example Vanderschuren, Acterberg, & Trezza, 2016). However, from a functional perspective, the difference we found between relaxed and competitive play suggests that they have different functions in the social life of wolves and highlights the need to consider play in its different components rather than just a unitary concept.

## Figures and Tables

**Table A1:** Behaviours used to determine agonistic and affiliative relationships outside the play context

Behaviour		Definition
**Rank indicator behaviours**		
Dominant behaviours		
	Stand tall	A straightens up to full height, with a rigid posture and tail; may include raised hackles, ears erect and tail perpendicular or above the back
	Stand over	A is standing over R's body, with all four paws on the ground, with the tail held high. R may have either the whole body or just the forepaws under A's belly/side
	No-play paw on	A places one or both forepaws on R's back; outside of the play context
	Ride up	A mounts R from behind or from the side, exhibiting a thrusting motion
	Head on	A approaches R's shoulder/back and puts its head on it. Formation usually looks like a capital ‘T’
	No-play muzzle bite	A grabs the muzzle of R either softly or with enough pressure to make the other whimper; outside of the play context
Submissive behaviours		
	Crouch	A lowers the head, sometimes bending the legs, arching the back, lowering the tail between the hindlegs and avoiding eye contact
	Passive submission	A lies on its back showing its stomach and holding its tail between its legs. The ears are held back and close to the head and A raises a hindleg for inguinal presentation
	Active submission	A has its tail tucked between the hindlegs sometimes wagging it while in a crouched position (with hindquarters lowered) and may attempt to paw and lick the side of R's muzzle. The behaviour may include urination
	Withdrawing	A withdraws from R moving away slowly in the opposite direction, displaying a submissive posture. It occurs when A has been threatened or attacked by R, or a fight has taken place
	Flee	A runs away from R with tail tucked between the legs and body ducked. It occurs when A has been threatened or attacked by R, or after a fight
	Avoidance	In response to R reducing the distance to it, A moves away displaying a submissive posture. A may also look at the individual it is trying to avoid
**Aggressive behaviours**		
	Threat	A orients towards R performing one or more of the following behaviours: staring at, curling of the lips, baring of the canines, raising the hackles, snarling, growling and barking, sometimes with the tail perpendicular or above the back
	Attack	A runs into or jumps onto R with tail, ears and sometimes hackles up, often with bites at the neck
	Knock down	A strikes R sharply with the chest or shoulder so that R falls to the ground
	Pin	A grabs R at the neck or muzzle, forcing it down to the ground and holding it there
	Fight	A and R engage in reciprocal biting and aggressive physical contact
	Chase	A runs after R, exhibiting threatening behaviours (see ‘threat’ above)
	Jaw spar	A and R ‘fencing’ with open jaws
	Snapping	A snaps its teeth into the air towards R
**Affiliative behaviours**		
	Grooming	A nips, licks or scratches the fur or skin, occasionally the neck of R
	Lie friendly	A lies on its back, tail-wags, sometimes kicking with the foreleg against R, often with open mouth
	Stand friendly	A stands with tail perpendicular to or below the plane of the back, wagging it, ears pointed forwards, while R is approaching it or orienting/looking towards it
	Social sniff	A sniffs R's body part except its anogenital area
	Body rubbing	A rubs its body against any part of R's body
	Body contact	A stays (for at least 10 s) with at least part of its body in contact with R; both A and R have a relaxed position

Adapted from the Wolf Science Center Social Behavior Ethogram. A: wolf performing the behaviour; R: wolf receiving the behaviour.

**Table A2:** Outputs from the model 1 analysis

Model	Variable	*df*	χ^2^	*P*
Full model	Competitive play duration	1	2.18	0.14
	Relaxed play duration	1	0.04	0.83
	Age combination	1	2.13	0.14
	Sex combination	2	2.21	0.33
	Kin relationship	1	8.69	0.003
	Competitive play duration*pack type	1	20.40	6.29e-06
	Relaxed play duration*pack type	1	7.06	0.007
	Pack type	1	20.40	2.29e-06
Pack type: ‘puppy’	Competitive play duration	1	12.34	0.0004
	Relaxed play duration	1	0.28	0.60
	Kin relationship	1	9.28	0.002
Pack type: ‘mixed-age’	Competitive play duration	1	10.14	0.001
	Relaxed play duration	1	5.34	0.021

Generalized mixed-effect model (GLMM) with a Poisson distribution, with the ‘frequency of aggressive behaviours’ as the response variable (normalized by observation time by including the offset function in the model formula), and ‘competitive play duration’, ‘relaxed play duration’, ‘age combination’ (e.g. ‘puppy–puppy’ versus ‘puppy–adult’ dyads), ‘sex combination’ (‘male–male’, ‘male–female’, ‘female–female’ dyads) and ‘kin relationship’ (‘kin’ versus ‘nonkin’ dyads) as predictor variables. The interactions between both competitive and relaxed play durations and ‘pack type’ were included. Statistics are given for each variable when they were last in the model.

**Table A3:** Outputs from the model 2 analysis

Model	Variable	*df*	χ^2^	*P*
Full model	Competitive play duration	1	0.48	0.49
	Relaxed play duration	1	9.38	0.002
	Age combination	1	0.75	0.39
	Sex combination	2	1.10	0.58
	Kin relationship	1	1.20	0.27
	Competitive play duration*pack type	1	1.83	0.18
	Relaxed play duration*pack type	1	5.02	0.025
	Pack type	1	2.00	0.16
Pack type: ‘puppy’	Relaxed play duration	1	3.21	0.07
Pack type: ‘mixed-age’	Relaxed play duration	1	9.03	0.003

Generalized mixed-effect model (GLMM) with a Poisson distribution, with the ‘frequency of affiliative behaviours’ as the response variable (normalized by observation time by including the offset function in the model formula), and ‘competitive play duration’, ‘relaxed play duration’, ‘age combination’ (e.g. ‘puppy–puppy’ versus ‘puppy–adult’ dyads), ‘sex combination’ (‘male–male’, ‘male–female’, ‘female–female’ dyads) and ‘kin relationship’ (‘kin’ versus ‘nonkin’ dyads) as predictor variables. The interactions between both competitive and relaxed play durations and ‘pack type’ were included. Statistics are given for each variable when they were last in the model.

**Table A4:** Outputs from the model 3 analysis

Variable	*df*	χ^2^	*P*
Frequency of competitive play behaviours	1	7.82	0.005
Age of the actor	1	85.10	< 2.2e-16
Sex of the actor	1	7.47	0.006
Age of the receiver	1	4.93	0.026
Sex of the receiver	1	0.40	0.52
Kin relationship	1	2.85	0.09

Generalized mixed-effect model (GLMM) with a Poisson distribution, with the ‘frequency of rank indicator behaviours’ (normalized by observation time by including the offset function in the model formula) as the response variable and the ‘frequency of competitive play behaviours’ (normalized by observation time), ‘age’ and ‘sex’ of both actors and receivers and ‘kin relationship’ as predictor variables. Statistics are given for each variable when they were last in the model.

**Table A5:** Outputs from the model 4a analysis

Variable	*df*	χ^2^	*P*
Dominance relationship asymmetry index	1	11.43	0.0007
Age combination	1	2.82	0.09
Sex combination	2	0.63	0.73
Kin relationship	1	6.37	0.012
Dominance relationship asymmetry index*pack type	1	0.18	0.67
Pack type	1	2.40	0.12

Linear mixed-effect model (LMM) with competitive play duration as the response variable and the ‘ramk asymmetry index’, ‘age’ and ‘sex combination’, ‘kin relationship’ as predictor variables. An interaction between the ‘rank asymmetry index’ and ‘pack type’ was included. Statistics are given for each variable when they were last in the model.

**Table A6:** Outputs from the model 4b analysis

Variable	*df*	χ^2^	*P*
Agonistic asymmetry index	1	0.34	0.56
Age combination	1	1.55	0.21
Sex combination	2	1.20	0.55
Kin relationship	1	0.21	0.64
Agonistic asymmetry index*pack type	1	0.26	0.61
Pack type	1	1.23	0.27

Linear mixed-effect model (LMM) with competitive play duration as the response variable and the ‘agonistic asymmetry index’, ‘age’ and ‘sex combination’, ‘kin relationship’ as predictor variables. An interaction between the ‘agonistic asymmetry index’ and ‘pack type’ was included. Statistics are given for each variable when they were last in the model.
